# Minus the Error: Estimating *d*_N_/*d*_S_ and Testing for Natural Selection in the Presence of Residual Alignment Errors

**DOI:** 10.1101/2024.11.13.620707

**Published:** 2024-11-15

**Authors:** Avery G Selberg, Maria Chikina, Tim Sackton, Spencer V Muse, Alexander G Lucaci, Steven Weaver, Anton Nekrutenko, Nathan Clark, Sergei L Kosakovsky Pond

**Affiliations:** 1 Institute for Genomics and Evolutionary Medicine, Temple University, Philadelphia, PA, USA; 2 Department of Biology, Temple University, Philadelphia, PA, USA.; 3 Department of Computational and Systems Biology, University of Pittsburgh, Pittsburgh, PA, USA.; 4 FAS Informatics Group, Harvard University, Cambridge, MA, USA.; 5 Bioinformatics Research Center, North Carolina State University, Raleigh, NC, USA.; 6 Department of Physiology and Biophysics, Weill Cornell Medicine, New York, NY, USA.; 7 Weill Cornell Medicine, The HRH Prince Alwaleed Bin Talal Bin Abdulaziz Alsaud Institute for Computational Biomedicine, New York, NY, USA.; 8 Department of Biochemistry and Molecular Biology, The Pennsylvania State University, University Park, PA, USA.

## Abstract

Errors in multiple sequence alignments (MSAs) are known to bias many comparative evolutionary methods. In the context of natural selection analyses, specifically codon evolutionary models, excessive rates of false positives result. A characteristic signature of error-driven findings is unrealistically high estimates of dN/dS (e.g., >100), affecting only a small fraction (e.g., ~0.1%) of the alignment. Despite the widespread use of codon models to assess alignment quality, their potential for error correction remains unexplored. We present BUSTED-E: a novel method designed to detect positive selection while concurrently identifying alignment errors. This method is a straightforward adaptation of the BUSTED flexible branch-site random effects model used to fit distributions of dN/dS, with an important modification: it integrates an “error-sink” component representing an abiological evolutionary regime (dN/dS > 100), and provides the option for masking errors in the MSA for downstream analyses. Statistical performance of BUSTED-E on data simulated without errors shows that there is a small loss of power, which can be mitigated by model averaged inference. Using four published empirical datasets, we show BUSTED-E reduces unrealistic rates of positive selection detection, often by an order of magnitude, and improves biological interpretability of results. BUSTED-E also detects errors that are largely distinct from other popular alignment cleaning tools (HMMCleaner and BMGE). Overall, BUSTED-E is a robust and scalable solution for improving the accuracy of evolutionary analyses in the presence of residual alignment errors, contributing to a more nuanced understanding of natural selection and adaptive evolution.

## Introduction.

In their 1994 paper introducing what is arguably the first modern tool for multiple sequence alignment (MSA), CLUSTAL-W, Thompson et al. wrote: “*...CLUSTALW will find an alignment which is difficult to improve by eye. In this sense, the alignment is optimal with regard to the alternative of manual alignment”* (Thompson et al. 1994). Today, consortia like Zoonomia and VGP are churning out hundreds of genomes, processed through incredibly complex bioinformatics pipelines, yielding, among other deliverables, tens of thousands of MSAs. The final MSAs are taken as error-free (with very few exceptions) input to various downstream analyses: phylogeny inference, conservation and evolutionary rate estimation, natural selection analyses, and many more. Even a suggestion of manual review of sequence data to check for errors strikes one as a quaint and wholly inadequate anachronism. Yet, no reasonable practitioner would suggest that these data are error-free or even nearly error-free. The frequency and nature of errors in massive genomic datasets are generally unknown, and for many methods used for evolutionary or biomedical analyses, their sensitivity to errors has not been systematically quantified, or assessed. Numerous papers do, however, show that errors matter a great deal qualitatively (Schneider et al. 2009; Markova-Raina and Petrov 2011).

**Figure 1.**
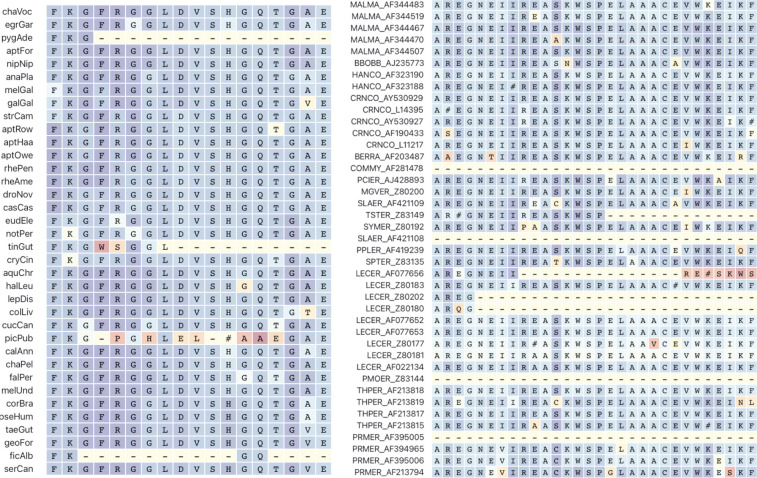
Two examples of local misalignment errors that are obvious to the eye (picPub sequence in the left panel, LECER_AF077656 sequence in the right). Redder colors indicate higher probability assigned to a particular codon by BUSTED-E, shown here as amino-acid translation, with # used to show ambiguous translations (e.g. codon ANC). The MSA on the left is the RAP1GAP2 alignment from birds and mammals, studied in a large scale screen from Shultz and Sackton (2019). The MSA on the right is for plant rbcL from Tamuri and Reis (2021).

Errors in MSAs are a combination of sequencing and assembly errors in the input sequences and errors in the alignment process itself. Since the 1990s, many iterative improvements to MSA generation and filtering have been proposed. Previous studies have produced methods to identify and filter specific types of errors or shown them to be unimportant (Landan and Graur 2007; Talavera and Castresana 2007; Sela et al. 2015). Continuing, and even accelerating rate of publications proposing new techniques for identifying and removing errors from MSAs (generally the inference of false homology) attests to the relevance and desirability of improved MSA quality. One can state with some confidence that the current state of the art ensures that gross or major MSA errors do not propagate downstream. But more subtle errors definitely do: see Figure 1 showing *prima facie* errors in alignments used in papers published in the previous decade.

The objective of this paper is to abrogate the effect of residual (post-filtering) errors in multiple sequence alignments (MSAs) on codon-based analyses of coding sequence evolution. Positive selection plays a crucial role in evolutionary biology, as it refers to the process where advantageous genetic mutations increase in frequency within a population. Specifically, in the context of *d*_N_/*d*_S_ estimation and testing for positive diversifying selection, researchers seek to identify regions in the genome where the rate of non-synonymous substitutions (*d*_N_) exceeds the rate of synonymous substitutions (*d*_S_). These analyses help to pinpoint areas under selective pressure that may contribute to an organism’s adaptive evolution. However, the presence of errors in MSAs can severely compromise these analyses.

Codon-based analyses can be very sensitive to events affecting a small fraction (<1%) of MSA rows and columns. High sensitivity is good when it retrieves subtle and transient signatures of selection, but disastrous when it hones in on data artifacts, particularly when those artifacts do not reflect underlying biological processes. Our objective here is not to replace alignment filtering methods but to enhance the robustness of downstream analyses by mitigating the impact of these residual errors. We aim to improve the reliability of detecting true signals of positive selection and diversifying positive selection, thereby advancing our understanding of evolutionary processes.

We rely on a simple empirically-derived intuition, namely that small alignment errors which become apparent upon inspection “by eye” tend to produce a specific pattern of parameter estimates in sensitive branch-site models (Murrell et al. 2015; Wisotsky et al. 2020). Specifically, these errors generate unrealistically high estimates of *d*_N_/*d*_S_ (*e.g.*, >100) which apply to unrealistically small fractions (*e.g.,* <1%) of the alignment. We show that a straightforward modification of the BUSTED family of methods to include an error-protection component, works remarkably well at noise reduction or elimination, and dramatically reduces unrealistic rates of positive selection detection in large-scale screens of MSAs, often by an order of magnitude, e.g. from ~40% to 5–10%. The reduced sets of genes inferred to be under selection tend to show stronger enrichment for functional categories expected to be under positive selection, and are less biased towards longer genes. This modification, which we call BUSTED-E (“minus the error”), does not require alignment pre-filtering, but deals with the error transparently. As a byproduct, BUSTED-E can annotate each alignment with locations of possible errors and mask them prior to the use of other tools which could be similarly affected by alignment errors.

## Results.

### A worked example.

To develop intuition for how BUSTED-E works, consider the ubiquitous task of deciding if a gene has been subject to diversifying positive selection using a *d*_N_/*d*_S_-based method. Having chosen a particular method (model and tool), a researcher estimates *d*_N_/*d*_S_ (ω) and uses a statistical test to assign a p-value to the hypothesis that ω > 1 on at least some subset of sites in the MSA of the gene and/or branches of the corresponding phylogenetic tree (species or gene).

We choose to work with the BUSTED-S method (Wisotsky et al. 2020), which estimates the ω distribution across sites with three classes (ω_1_, ω_2_, and ω_3_), and where the evolution along each branch at each site is described by a random independent draw from this distribution, and where only one value (ω_3_), is permitted to take values ≥1, i.e. positive diversifying selection for amino acid replacement. BUSTED-S uses a likelihood ratio test to compute a p-value versus the null hypothesis where ω_3_ ≤ 1. Other models frequently used in the literature include the site or branch-site models implemented in the PAML software suite (Yang 2007) or Bayesian approaches (Murrell et al. 2013; Rodrigue and Lartillot 2014). The BUSTED framework can accommodate flexible rate distributions, while being computationally tractable and scalable (Kosakovsky Pond et al. 2011). The BUSTED-E method extends BUSTED-S by adding an evolutionary category described by ω_E_ ≥ 100, whose maximal weight is limited by 1%. This is an “error-sink” category, which is meant to capture the (false) evolutionary signal derived from local alignment errors like those shown in Figure 1. The limits on the ω_E_ category are heuristic and can be tuned, but as we discuss in a later section, these values have been inspired by examining patterns of parameter estimates in large collections of alignments.

To illustrate the new model we will use two genes from the 11,267 analyzed for evidence of selection by Shultz and Sackton (2019). Seeking to quantify positive selection across bird and mammalian genomes, this work used a sophisticated pipeline for data collection, curation, and analysis including multiple filtering steps for the MSAs and the use of multiple positive selection inference methods. BUSTED-S finds high statistical significance (p < 0.001) that both of these genes are subject to episodic diversifying selection (Table 1). For gene 37542 (HAUS augmin-like complex subunit 1, HAUS1), the “positive selection” component has a moderate ω_3_=2.27 with broad support (29.3% of the alignment), but for gene 24389 (peroxidasin like, PXDNL), the same component has a large ω_3_>1000 with narrow support (0.01% of the alignment). Because the selection signal is based on an implausibly small subset of codons and because the inferred value for ω is implausibly large, this finding could be due to error. Manual MSA inspection reveals that this is likely due to a local misalignment of several sequences in the 3’ end, which results in several apparent multi-nucleotide substitutions and several codon sites with low homology (Figure S1).

BUSTED-E estimates the same three ω classes, with the addition of the error class, ω_E_, which is forced to have ω≥100 and cannot draw more than 1% weight. This leads to a dramatic reversal in support for episodic diversifying selection (EDS) for PXDNL (24389), where all of the apparent positive selection weight is absorbed by the error class, and the rest of the distribution is accordingly affected. HAUS1 (37524) maintains a positive selection classification, even though there is also a non-zero error component, i.e., selection is present but so may be errors.

### Reanalysis of genome-wide selection scans.

To understand the prevalence of misalignment errors and the impact they may have on selection detection analyses, we selected four large-scale suitable published studies (Schneider et al. 2009; Nguyen et al. 2015; Wu et al. 2018; Sackton 2020) whose authors made their alignments publicly available (Table 2). All four studies made use of one or more codon-based *d*_N_/*d*_S_ tests (as implemented in HyPhy and/or PAML) to examine a thousand or more coding sequence analyses for evidence of positive selection. All of the papers performed some automated and (often) additional manual quality control and filtering of their alignments prior to subjecting them to *d*_N_/*d*_S_ analysis, reflecting a field-wide appreciation that such filtering is necessary. As it is impractical and generally irreproducible to curate and screen thousands of alignments by hand, all studies relied on automated quality control and filtering, a practice common in the field, and a key target area for applications for BUSTED-E. The studies spanned a chronological decade, were done by unconnected research groups, varied in taxonomic range and alignment size, and differed in research goals. All original and filtered multiple sequence alignments and trees are available from https://github.com/veg/pub-data

#### BUSTED-E drastically reduces the rate of likely erroneous positive selection detection.

**Figure 2.**
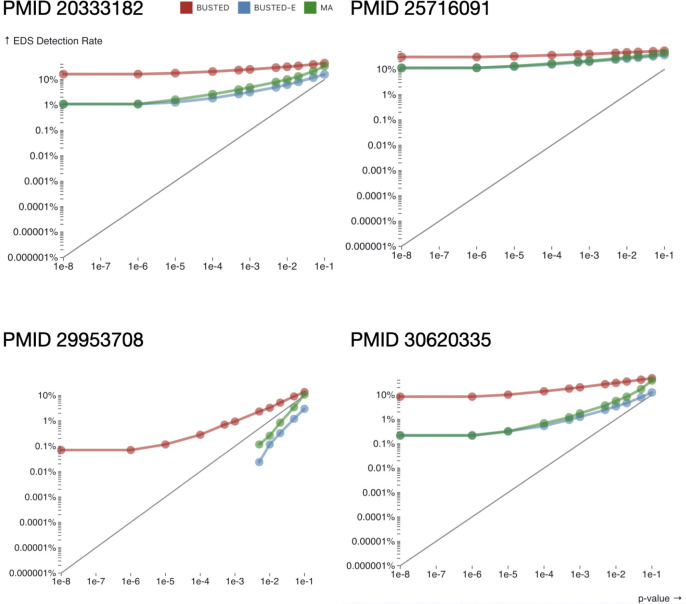
The fraction of alignments detected as being subject to episodic diversifying selection (Y-axis) as a function of testing stringency (significance level, X-axis); MA – model averaged results. The gray line depicts nominal detection rates, i.e., rates expected when only null/neutral data are present.

When we compared the rates of detection for episodic diversifying selection (EDS), using the standard BUSTED (Wisotsky et al. 2020) model with synonymous rate variation, and the error-compensated BUSTED-E model, the latter returned many fewer positive results (Table 3), across the range of test stringencies (Figure 2). Even when using the less conservative model averaged approach (MA), where results from BUSTED-S and BUSTED-E models are combined using goodness-of-fit weighting (to mitigate the potential loss of power, see Methods), the rate of detection is lowered substantially, especially for smaller p-value cutoffs. Note that if only a small fraction of alignments are truly subject to positive selection, then the fraction of alignments found to be significant should actually be close to the chosen significance level (*e.g.,* when using a significance level of 0.01 we probably shouldn’t see many more than 1% of alignments identified as positively selected, since 0.01 is the expected false positive rate of the test). In Figure 2 we see that observed significance rates for error-corrected models appear to be more in line with this expectation. Remarkably, three of the four datasets have a sizable fraction (~10–30%) of alignments where BUSTED-S detects EDS even for very stringent significance p-value cutoffs. We hypothesized that BUSTED was “certain” of EDS due to the presence of anomalous alignment regions in many of them. Only one of the four previous studies included directly comparable results (BUSTED in Shultz and Sackton (2019), detection rate 0.45). For the other two studies where *d*_N_/*d*_S_ > 1 tests were done, the uncorrected BUSTED-E detection rate was similar to or lower than the rates obtained following multiple testing correction. However, it should be noted that these studies used branch-site tests focusing on specific lineages or clades, whereas here screen for selection on the entire alignment.

#### Datasets where BUSTED and BUSTED-E disagree have a characteristic pattern of inferred rates.

**Figure 3.**
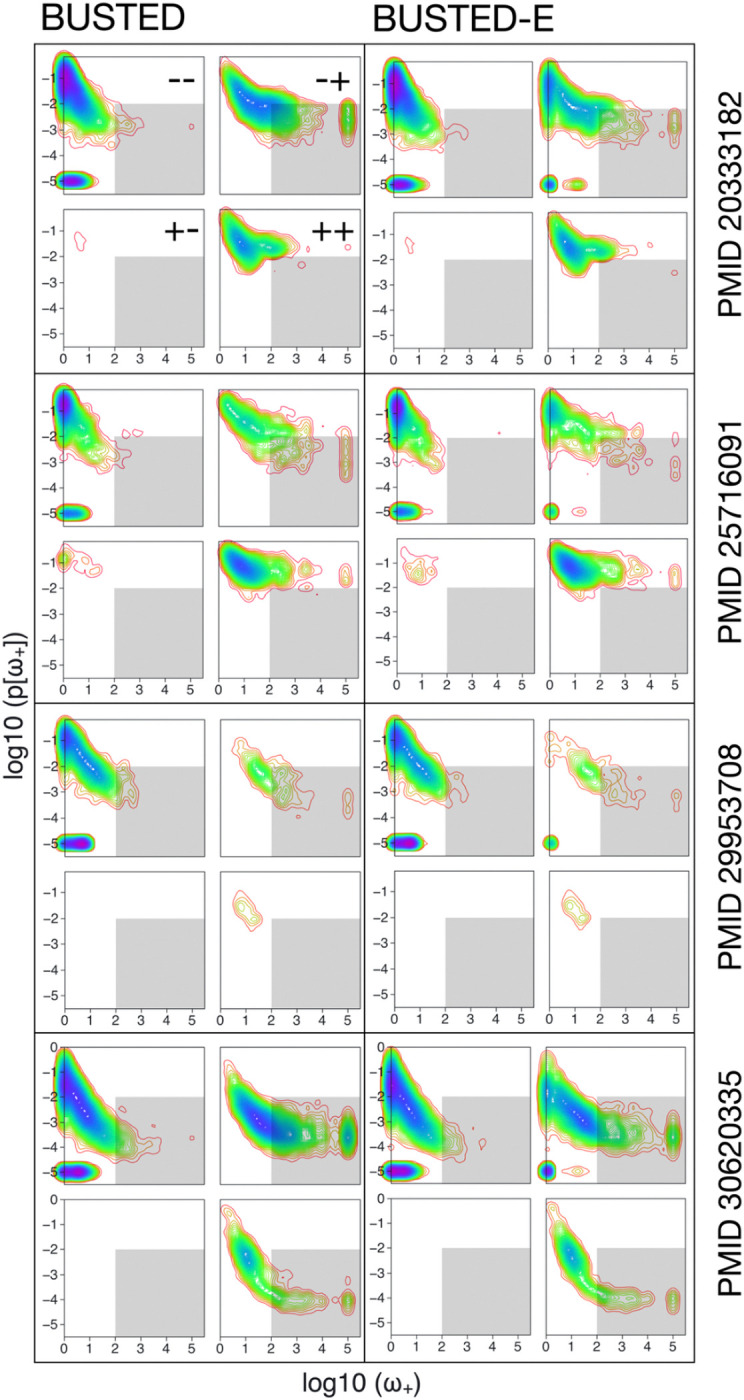
Contour plots of ω≥1 (ω_+_) and corresponding weight estimates stratified by model/dataset/concordance. The +/− designation refers to whether or not BUSTED (first character) and BUSTED-E (second character) classify the datasets as subject to EDS (+) or not subject to EDS (−). The grey square in the bottom right depicts the “twilight zone”: ω_+_≥100, p[ω_+_] ≤0.01.

Many of the datasets where BUSTED and BUSTED-E disagree on the presence of EDS (Figure 3, −+ and +− categories) have a characteristic shift in the estimates of both the magnitudes of the ω≥1 component and the weight associated with it to the “twilight zone”. In this zone, the model allocates less weight (≤1%) to larger values of ω (≥100). This clear pattern bolsters the intuition for how we chose to parameterize BUSTED-E. If the positive selection component has a very large ω value with a very low weight it could very well be due to a local misalignment or non-homology error. Indeed, when the two models agree, the corresponding distribution of estimated rates and weights is much more similar between BUSTED and BUSTED-E. For the positive selection component, BUSTED-E yields significantly lower estimates of ω across all datasets (Table 2).

#### The inferred error component is small.

Across all four datasets, BUSTED-E allocates a mixture weight well below 1% on average, typically less than 0.1% (Table 3). The majority of the alignments (with one exception) have an estimated error weight of exactly 0, *i.e.,* the BUSTED-E model effectively reduces to the simpler BUSTED model. As shown in a later section, a median of 9–32 individual codons per alignment (Table 8) are marked for masking by BUSTED-E.

Relatively few alignments have a strict statistical preference for BUSTED-E. While BUSTED is nested within BUSTED-E, it is not “properly nested” in the sense that BUSTED-E simplifies to BUSTED either by setting the weight of ω_E_ to 0 or by setting ω_E_=ω_3_. Thus, while the two models should not be compared on a particular alignment via a likelihood ratio test with a single degree of freedom, a conservative likelihood ratio test with two degrees of freedom is appropriate (Self and Liang 1987; Murrell et al. 2015). For the four datasets in Table 3, between 0.5% and 6.9% of the alignments reject BUSTED in favor of BUSTED-E (p≤0.05), with 24389/PXDN from Table 1 serving as an example. For these datasets, the presence of an error component provides a significantly better fit to the data. However, on the majority of alignments where the two models disagree (BUSTED finds evidence of EDS, and BUSTED-E does not), BUSTED cannot be rejected in favor of BUSTED-E. For the Shultz and Sackton (2019) dataset, for example, of the 3,814 alignments where BUSTED finds EDS (at p = 0.05), there are 3,552 (93.2%) cases when BUSTED cannot be rejected in favor of BUSTED-E (at p = 0.05). We examine main reasons for discordant alignment classification next.

### Better performance of the BUSTED-E null (no EDS, with the error component) explains much of the discordance between the models.

Unlike BUSTED, where the null model for EDS testing does not include any provision for positive selection (all ω≤1), the error component with ω_E_≥100 is still available for BUSTED-E. In other words, the null model (no EDS) under BUSTED-E can reallocate some of the mixture weight to this error class. To illustrate, we first consider alignment SUGP2 (10004) from Shultz and Sackton (2019) (Table 4), where BUSTED shows strong evidence for EDS (p < 0.0001), but BUSTED-E does not (p=0.12). The alternative models for EDS tests have exactly the same log likelihood scores and rate estimates (there is no error estimated component), but for the null model, BUSTED-E allocates 0.05% weight to the error component. The specific sites which are most affected by this change are those where multi-nucleotide mutations/substitutions (MNMs, e.g. CAT : AGA) are inferred to occur. Several recent studies lay out compelling evidence that MNMs do indeed affect tests for positive selection, most notably through increasing false positive rates when models that do not allow them are used (Venkat et al. 2018; Dunn et al. 2019; Lucaci et al. 2023). BUSTED-E can be extended to model MNMs directly (discussed below), although modeling MNMs is not our main focus here. Second, consider another discordantly classified alignment 11294 from Shultz and Sackton (2019) (Table 5) Here, both BUSTED (EDS p < 0.0001) and BUSTED-E (EDS p = 0.5) estimate large ω_**3**_ values for very small fractions of otherwise highly conserved alignments, which obviates the need of a separate error component for unrestricted BUSTED-E. However, the BUSTED-E null model can simply absorb this high rate in the error class, while constraining ω_**3**_≤1. The few sites where BUSTED derives the selection signal from are also enriched for MNM.

#### Qualitative categorization of discordant alignments.

We bin all alignments where BUSTED returns a positive EDS classification, and BUSTED-E does not into five qualitative categories.

1. Statistically supported error component, defined as a significant LRT test which rejects BUSTED in factor BUSTED-E. These are the alignments that should be the primary focus for error filtering, because they contain selection signal (non-empty positive selection ω_**3**_ component in BUSTED) and an error signal (non-empty and statistically supported error ω_**E**_ component in BUSTED-E), *e.g.*, gene PXDNL from Table 1.
2. Possible model misspecification, defined as having an empty error class (weight of ω_**E**_ is 0), a positive selection component (ω_**3**_**<100)**, and an insignificant BUSTED v BUSTED-E LRT test. For these alignments all the selection signal from BUSTED is absorbed by the error class in BUSTED-E null (*e.g.*, gene SUGP2, Table 4). Thus, the evolutionary process giving rise to substitution patterns interpreted as EDS by BUSTED generates abiological substitution rates which may instead reflect model inadequacy.
3. Only abiological selection rates, defined as having an empty error class (weight of ω_**E**_ is 0), a positive selection component **(**ω_**3**_**≥100**), and an insignificant BUSTED v BUSTED-E LRT test. For these alignments there is no statistical support for anything other than an abiologically high evolutionary rate (e.g., gene COPB1, Table 5).
4. Weak basis of selection support, defined as having a non-empty error class (weight of ω_**E**_ is > 0), a small (<0.01) weight for the selection component **(**ω_**3**_**≥100**) in BUSTED, and an insignificant BUSTED v BUSTED-E LRT test. These datasets are similar to class type 1 defined above, but fail to reject BUSTED vs BUSTED-E.
5. All others, alignments that do not belong to any of the previous categories. These include borderline cases (BUSTED p-value near the significant thresholds), or alignments where BUSTED-E may have suffered power loss (see the simulation section).

We can classify alignments as discordant either when BUSTED and BUSTED-E disagree, or when BUSTED and the model averaged (MA) result disagree. Using MA as the alternative to BUSTED is more conservative: the overall number of discordant results decreases, as does the proportion of Type 2 disagreements.

### Power loss suffered by BUSTED-E on null data is not sufficient to explain reduced detection rates.

**Figure 4.**
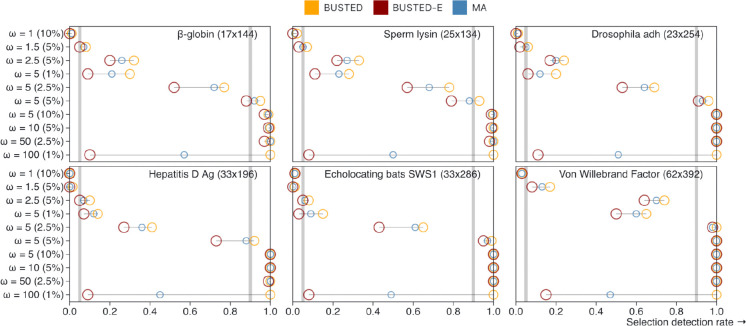
Rates of EDS selection on data simulated with the BUSTED model. A LRT test with p≤0.05 constitutes a positive result. Horizontal reference lines demarcate 0.05 and 0.90 rates. The datasets are sorted by the number of characters, smallest to largest. Circles are of different sizes to eliminate overlap.

To gauge the extent of power loss and possible parameter estimation bias, we analyzed 6,000 alignments simulated parametrically under the BUSTED (no error class) model using 60 different model configurations (see Methods). The results are summarized in Figure 4 and shown in greater detail in Figure S2. For any simulation setting, BUSTED≥MA≥BUSTED-E power relationship holds. This is expected, because on data where the true generating model is BUSTED, BUSTED-E should be no more efficient, and model averaging will interpolate the power between BUSTED and BUSTED-E. We also define the relative power loss, L_p_ (model1, model2) = power (model2) / power (model1). The following observations can be made:

1. For the scenario without EDS [ω = 1 (10%)] all models had false positive detection rate of less than 5% at p = 0.05. The addition of the error class does not inflate the false positive rate.
2. For the scenario that mimics the error class [ω = 100 (1%)] the expected behavior is observed. The BUSTED model has ~100% power and the BUSTED-E model has <10% power because the error component captures most of the variation in the error class. Using model averaging, the power settles around 50%. In other words, if simulated data have EDS that is misinterpreted as the error class (very high ω but at low proportion), BUSTED-E will miss it.
3. For scenarios where EDS is strong and wide-spread [ω = 5 (10%), ω = 10 (5%), and the very high ω = 50 (2.5%)], BUSTED has power ≥99%, BUSTED-E has power ≥97%, and MA : power ≥99%. In particular, BUSTED-E does not absorb ω = 50 in the error class.
4. For the ω = 5 (5%) scenario, a more nuanced behavior can be observed. For sufficiently large/diverse datasets (*adh*, *SWS1*, *vwf*), all methods have power ≥90%, but the power drops below 90% for other datasets. L_p_ (BUSTED-E, BUSTED) ranges from 0.79 to 1.0 relative, and L_p_ (MA, BUSTED) - in 0.94 to 1.0 relative power. For example, on the Hepatitis D Ag dataset, detection rates are 92% (BUSTED), 73% (BUSTED-E), and 88% (MA). These numbers are 95%, 88%, and 92% for β-globin.
5. As simulated EDS strength and pervasiveness drop, power degrades for all of the methods, with larger datasets having higher values for a given ω and weight combination. For very weak selection [ω = 1.5 (5%)], the maximum power does not exceed 17% (BUSTED, *vwf*) for any method. The most interesting cases occur for intermediate selection strength settings, e.g., ω = 5 (1%) or ω = 5 (2.5%). Here, the absolute difference between BUSTED and BUSTED-E power rates is the greatest, even though for most data sets BUSTED has at best moderate (<50%) power. For ω = 5 (2.5%), L_p_ (BUSTED-E, BUSTED)≥ 0.675 and L_p_ (MA, BUSTED)≥ 0.87. For ω = 5 (1%), L_p_ (BUSTED-E, BUSTED)≥ 0.2 and L_p_ (MA, BUSTED)≥ 0.6. However, when BUSTED-E suffers significant power losses compared to BUSTED, BUSTED has low detection rates to begin with: e.g., 15% (BUSTED) vs 3% (BUSTED-E) vs 9% (MA) for SWS at ω = 5 (1%).
6. Excluding the datasets simulated under the regime consistent with the error class assumptions [ω = 100 (1%)], and two *vwf* dataset scenarios, the rate at which BUSTED was rejected in favor of BUSTED-E did not exceed 5%. Because rejection of the correct model constitutes a false positive, this behavior is expected and desirable. Two *vwf* scenarios had elevated rejection rates: 6% for ω = 10 (5%), and 12% for ω = 50 (2.5%). For the 12 datasets where BUSTED is rejected in favor of BUSTED-E [ω = 50 (2.5%)], the latter underestimates true ω: mean of 30.6 vs 50.4 for the remaining 88 datasets.
7. There is no evidence of parameter estimation biases specific to BUSTED-E, except for the ω = 100 (1%) scenario, as expected. As shown in Figure S2, BUSTED, BUSTED-E and MA approaches all yield very similar interquartile ranges for ω and weight parameter estimates. In some cases, all models exhibit dataset and parameter-combination specific biases, *e.g.*, in underestimating ω and overestimating its weight under the ω = 5 (1%) scenario for all datasets except β-globin. These dataset-specific empirical biases are not specific to the model with the error component.

Our simulations suggest that while BUSTED-E does suffer some power loss when the underlying data are generated using a model without the error component, this power loss is minimal for cases where the selection effect is strong (ω and/or its weight are high). BUSTED-E shows a more significant power loss in the cases when selection is weaker, but that also occurs with BUSTED. Model averaging is able to recover much of the power loss due to the unnecessary error component for all scenarios. Finally, BUSTED-E does not introduce additional systematic biases to parameter estimates. Overall, we conclude that the dramatic reduction in EDS detection rate cannot be primarily attributed to power loss.

### BUSTED-E reduces bias in positive selection calls, improving biological interpretation.

Because ground truth in selection analyses is not known, we cannot claim that our method is provably better for detection of positive selection. Instead, we evaluated the implications for genome-wide analysis of positive selection signal distribution. We hypothesized that while protein length is a key determinant of detection power for positive selection, as it directly relates to sample size, longer proteins are, on average, more prone to alignment errors. Therefore, we predict that the BUSTED-E model should mitigate this length-related bias. To test this, we focused on the top 5th percentile of genes ranked by the value of the likelihood ratio test statistic (LRT) for both BUSTED and BUSTED-E models in the Schulz and Sackton (2019) dataset. We plot the distribution of these top-ranked genes across length bins, each containing an equal number of genes from the entire dataset (Figure 5, panel A). In a scenario without length bias, the number of top-ranking EDS genes should be approximately equal for each gene length. Instead, we observe an enrichment for longer genes detected with EDS by both models, as expected, given the relationship between length and statistical power. However, BUSTED-E markedly reduces the length bias seen in BUSTED alone, both by having more datasets in the shorter bins, and fewer datasets in the longest bins.

A central question in any genome-wide analysis is identifying the specific evolutionary pressures driving positive selection signals. We ran enrichment analyses using a collection of functional annotation sources (KEGG, Reactome, GO, and WikiPathways) on the same top 5% of genes (Figure 5B). Analyzing a combined set of pathways with FDR < 0.2 in either set, we find that BUSTED-E and BUSTED highlight different pathways with slightly more pathways (10 vs 8) being more enriched in BUSTED-E. However, the results obtains by BUSTED-E are much more closely aligned with both the original Schulz and Sackton analyses, which made a consensus call of several methods to identify positively selected genes, and other analyses highlighting the general overrepresentation of immune genes among the positively selected set (Vinkler et al. 2023). An analysis that more closely replicated the original workflow (Figure S3), using p≤0.05 to select the gene set for enrichment testing showed that under BUSTED-E more pathways (GO and KEGG) were enriched for selected genes, and that these pathways were in line with biological expectations (immune and pathogen interacting genes), compared to similar results using BUSTED. Such findings are also consistent with the central role of host pathogen interactions in driving diversifying selection (Enard et al. 2016). On the other hand, pathways preferentially enriched in BUSTED have little biological coherence or alignment with prior knowledge.

Further analysis suggests factors other than the action of positive selection may contribute to EDS detection with BUSTED. Specifically we observe that for pathways preferentially enriched in BUSTED the genes driving the enrichment tend to be unusually long. To quantify the overall trend we color-code each pathway according to how well protein length predicts whether a gene is part of the foreground set identified by the method showing the highest enrichment for that pathway. We quantify the association via a rank-based AUC metric, where a value of 1 would indicate that the foreground pathway genes are ranked entirely at the top by length while a value of 0.5 indicated a random length distribution. This approach reveals that for pathways specific to BUSTED the genes contributing most to the pathway enrichment often exhibit substantial length bias, i.e., longer proteins may disproportionately drive enrichment signals. For example, considering the top BUSTED pathway of “Microtubule-Based Transport” (GO:0099111) the median length for the foreground gene is 1593 codons while the overall median length in the dataset is 390. In contrast the pathways highlighted by BUSTED-E have relatively weaker length bias with lower length AUCs.

### Characterizing BUSTED-E alignment filtering.

BUSTED-E can be used to annotate individual codons in the input alignment with a numerical score which corresponds to the confidence that any particular codon is attributable to the error class (see Methods). Codons with sufficiently high error-class scores may then be masked with gaps and passed on as input to other analytical tools. As an illustration, consider HAUS1 and PXDNL genes from Table 1. The filtering procedure masks 90 codons (0 to 6 per sequence) from the HAUS1 alignment, and 42 codons (0 to 5 per sequence) from the PXDNL alignment. Examples of filtered codon sites and specific criteria for selecting sites to filter are provided in Methods (e.g., see Figure 12). BUSTED-S and BUSTED-E agree on the classification of both filtered alignments, whereas they disagreed on the original PXDNL alignment. Distribution parameter estimates are also much more similar when filtered datasets are used. Notably, even following the first pass of filtering, there is some residual error signal for the PXDNL gene, although the fraction allocated to it has been reduced by ten-fold.

When applied to the empirical alignment collections, BUSTED-E filtering masks a rather modest fraction of input alignments (Table 8). For the substantial majority of alignments, the filtering procedure does nothing. For the alignments where at least one codon is filtered, an average of 2–3 codons per 1000 codons are masked. With the exception of Wu et al. (2018) (gapless input alignments), the filtering procedure adds 5–10% of the total gaps in the resulting filtered alignments. In other words, one newly masked codon is added per 10–20 gapped codon positions already present in the input alignment.

Next, consider the effect that filtering has on selection detection with the standard BUSTED (no error component) models (Table 3). For all four datasets, when BUSTED is applied to filtered datasets, EDS detection rate is reduced significantly but remains higher than BUSTED-E on original datasets in three out of four cases, with the exception of Wu et al. (2018). In these three cases, BUSTED-E and MA approaches have essentially the same detection rate on original/filtered data—an indication of internal consistency. The dissenting dataset has an unusually high fraction of original alignments where BUSTED-E is preferred to BUSTED by LRT (0.2), and these alignments are gappy (~5% of total alignment size comprises gaps, see Table 2). Indeed, masking the codons most significantly contributing to the error signal in the original data reduces the residual ω_**E**_ weight (last column of Table 3) several fold, with >90% of the datasets allocating 0 weight to this model component.

### Evaluating the performance of BUSTED-E filtering alongside existing alignment filtering methods

We applied two widely used alignment filtering methods: BMGE (Criscuolo and Gribaldo 2010) and HMMCleaner (Di Franco et al. 2019) to alignments from Schulz and Sackton (2019), and compared how they performed relative to BUSTED-E. The primary goal was to determine whether BUSTED-E targets the same error modalities as the existing methods, both of which are motivated by finding regions of sequences that have low homology to other sequences. The results of this comparison are summarized in Table 9.

1. HMMCleaner and BMGE target different errors than BUSTED-E. This follows from (a) noticing that BUSTED found EDS at about the same rate (~30–40%) in the alignments filtered by these methods as in the original alignments, and (b) because EDS detection with BUSTED-E was dramatically lower than BUSTED for datasets passed through HMMCleaner and BMGE. In other words, from the standpoint of BUSTED-E, most of the errors it detects pass unchallenged by the other two tools.
2. HMMCleaner, BMGE, and BUSTED-E all mask very small fractions of alignments (0.02–0.2%).
3. The average run times for BUSTED-E, which performs selection detection and error-filtering jointly, are comparable to the average run times of HMMCleaner and BMGE, which only perform filtering.

It is important to reiterate that Schulz and Sackton (2019) alignments had already undergone alignment filtering with custom scripts prior to their publication (Jarvis et al. 2014). Consequently, it is reasonable to assume that most of the homology, annotation, or assembly errors which are the primary targets of BMGE and HMMCleaner had already been removed and what remains are second-order, smaller errors that affect individual sites.

### BUSTED-E model extensions.

The core idea of BUSTED-E is to identify alignment regions which appear aberrant in relation to the underlying evolutionary model. When this model is changed, the definition of aberrant patterns is also likely to change. Consider, for example, what happens if the codon substitution model is extended to permit instantaneous multi-nucleotide (MH) substitutions (Lucaci et al. 2021). As we and others have shown previously (Lucaci et al. 2023), including MH support in the context of selection detection dramatically lowers the rate of detection. For example, on data from Schulz and Sackton (2019), MH-enabled models detected EDS at a rate about 3 times lower than models without MH support (Lucaci et al. 2021). Much of this reduction is attributable to the difference in how multi-nucleotide substitutions that occur along short branches are handled. For standard models, MH substitutions drive up the estimates of ω, and in many cases one or a few of these substitutions is all that lends statistical support to positive selection in an alignment. For MH-enabled models these types of substitutions will be accommodated by separate 2- or 3-hit substitution rates, and reduce ω estimates. As an illustration, we include examples of datasets which are discordant under BUSTED and BUSTED-E (Table 6), but not discordant under +MH versions of this model from each of the five qualitative categories we defined previously. Thus, multihit and error model components are interacting, with the change in the baseline model substantively modifying what data features are considered “error-like”.

We reran the alignments from Schulz and Sackton (2019) with the BUSTED-MH model as the baseline, adding the error-sink component, and made the following observations.

1. The inclusion of +MH reduces EDS detection rates about four-fold, to 10.1% with BUSTED and to only 1.8% with BUSTED-E.
2. The datasets where BUSTED (without MH) finds EDS and BUSTED-E (also without MH) does not, are somewhat enriched for datasets where there is statistical evidence for including MH support (Odds Ratio = 1.2, p < 0.001). The addition of MH support to BUSTED abrogates EDS detection rates. For example, ~20% of the datasets assigned to Type 2 discordant class (“Possible model misspecification”) are also discordant between BUSTED and BUSTED-MH, with discordant datasets showing elevated rates of multi-nucleotide substitutions compared to the negative datasets (Figure 5). Interestingly, concordant positive datasets also have MH rates elevated compared to concordant negative datasets (Figure 5).
3. There is an interaction between MH rates and the presence of the error component; when a non-zero error component is inferred, 2- and 3-nucleotide rate estimates are lowered (Table 10).
4. There is a significant additional computational cost for adding the -MH component. On average, BUSTED-MH run times are on average 8x longer than BUSTED run times, and BUSTED-MH-E run times are on average 9x longer than BUSTED-E run times.

## Methods.

### Model description.

BUSTED-E (**B**ranch-site **U**nre**s**tricted **T**est for **E**pisodic **D**iversification with an **E**rror component), is a random-effects model of codon evolution, which builds upon the BUSTED class of models (Murrell et al. 2015; Wisotsky et al. 2020; Lucaci et al. 2023). BUSTED-E is designed to test for a presence of a fraction of the alignment evolving with *d*_N_/*d*_S_ (ω) > 1, i.e. subject to diversifying positive selection, while also accommodating an “abiological”, or error-like, mode of evolution. The key parameters describing the evolution at alignment site **s,** along the branch of the branch of a phylogenetic tree **b** are the instantaneous rate of synonymous substitutions **ɑ** and the ratio of non-synonymous to synonymous substitutions ω, respectively.

The key feature of BUSTED-E, is the inclusion on an “error-component” to the distribution of ω, with its ratio value bounded below by a large (a priori specified) number E, e.g. E = 100, and the maximal weight that can be allocated to this error component bounded from above by pe, e.g. 1%. The simple institution is that exceedingly large ω values are a hallmark of “fatal” deviations from the expected evolutionary process, and as such are categorized as due to alignment or sequencing error. We chose these specific values because they represented specific modes of error in empirical data (Figure 3), and because they were decimal “round” numbers. Error class parameters can be adjusted, of course. In all other respects, BUSTED-E is identical to the previously described and validated BUSTED[S] model. The default (but user-tunable) hyperparameter values are N=4 (number of ω bins) and M=3 (number of **ɑ** bins). For N=4, the ω distribution accommodates two classes for negative selection or neutral evolution (ω_**1**_ **≤** ω_**2**_ **< 1**), one class for positive selection or neutral evolution (**1 ≤** ω_2_), and one class for “errors” (**100 = E ≤** ω_**3**_).

### Formal model definition.

The BUSTED-E model is a standard continuous time discrete space Markov model of codon substitution, which partitions codon substitutions into three classes, with the corresponding instantaneous rate matrix Q, parameterized following the Muse-Gaut (MG94) (Muse and Gaut 1994) approach.

Here, $\theta_{ij}=\theta_{ji}$ denote five estimated nucleotide level substitution bias parameters ($\theta_{AG}=1$ for identifiability), $\pi_{j}^{k}$ are the cF3x4 estimates of position-specific equilibrium frequencies for the target nucleotide of the substitution (Pond et al. 2010). As described above, **ɑ,** ω vary from site-to-site and from (branch,site) to (branch,site), respectively. All parameters, including branch lengths, are estimated using maximum likelihood, as implemented in the HyPhy package v2.5.56 or later (Kosakovsky Pond et al. 2020).

### Statistical testing using BUSTED-E.

As noted above BUSTED-S is not properly nested within BUSTED-E since BUSTED-S can be obtained by constraining either ω_**E**_ or its weight to a boundary value. Therefore, we use the conservative $\chi^{2}$ distribution with two degrees of freedom to compute LRT p-values for rejecting BUSTED[S] in favor of BUSTED-E.

We test the alignment for evidence of episodic diversifying selection (EDS) by constraining ω_**N-1**_**=1**, optimizing this null model, and comparing it to the alternative BUSTED-E (where ω_**N-1**_**≥1**) using the likelihood ratio test. Once again, we use the $\chi^{2}$ with two degrees of freedom as the asymptotic distribution of the test statistic to assess significance.

Because the addition of an error rate class is expected to reduce statistical power (see simulations) when the correct model has no error component, or when the alignment is “error-free”, we compute a model-averaged test of significance (following the general framework described in (Lucaci et al. 2023). To do so, we fit both BUSTED[S] and BUSTED-E to the same data, compute the Akaike weights for each model using the small sample AIC_c_ information criterion score, and weigh LRT p-values for EDS by the corresponding Akaike weights: **p**(MA) = **w**(BUSTED[S])**p**(BUSTED[S]) + **w**(BUSTED-E)**p**(BUSTED-E). An Akaike weight of the model is defined as ~**exp** 0.5(min AIC_c_ − model AIC_c_), with the minimum taken over the models being compared, and normalized to sum to 1 over all models.

Model averaging is a way to control potential power loss. Assuming a worst case scenario, where BUSTED-E offers no improvement in log likelihood at the cost of two additional parameters, its AIC_c_ score will be 4 or more units higher than the AIC_c_ score for BUSTED[S]. Further assume that **p**(BUSTED-E) = 0.5 (the highest p-value for one-sided tests as defined in BUSTED). The contribution from BUSTED-E to the model averaged p-value will be 0.5 × **w**(BUSTED-E) ≤ 0.06.

### Model variations.

The BUSTED-E concept can be extended in several directions. First, we could alter the baseline model, for example by removing the synonymous rate variation (SRV) component, or adding a component for accommodating multi-nucleotide substitutions (Lucaci et al. 2023). Because the underlying model specifies what the expected biological reality is, it will influence the inference of what constitutes possible alignment errors. Second, we could partition the tree into “foreground” and “background” branches, as is commonly done for detecting lineage-specific selection, and apply to them different ω distributions and error components.

### Heuristic error filtering.

Given a BUSTED-E model fit, we implement the following heuristic procedure for alignment filtering. Our procedure applies to codons at individual branches, and consists of “masking” a subset of codons with gap characters (---). First, for each branch and site in the alignment, we compute two empirical Bayes factors (EBF) asking 1) if the ω value comes from the error class (EBF_e_), and 2) if the ω value comes from the (non-error) positive selection class (EBF_s_). To obtain those EBFs, we compute, for each (branch, site) pair **N** conditional probabilities $\mathrm{Pr}(\omega =\omega_{k}|branch,\;site,\;\hat{\Theta }),k=1\ldots N$, with $\hat{\Theta }$ used to represent all other parameter estimates. This involves the calculating N site-level phylogenetic likelihood functions, setting the weight of $\omega_{k}$ to 1 on a particular branch. These conditional probabilities can be converted into empirical Bayes posterior probabilities, simply by dividing each by $\Sigma_{k}\mathrm{Pr}(\omega =\omega_{k}|branch,\;site,\;\hat{\Theta })$ and computing EBF as the ratio of posterior to prior odds of $\omega =\omega_{k}$. The computational cost of the procedure for all (branch, site) pairs is roughly equivalent to **N** full-alignment likelihood calculations, and is negligible compared to the cost of BUSTED-E model fitting. EBF_e_ is computed for the events $\omega =\omega_{N}$ (error class) vs $\omega \neq \omega_{k}$ (not error class). EBF_s_ is computed for the events $\omega =\omega_{N}$ (error class) vs $\omega =\omega_{N-1}$ (selection class).

A (branch, site) will be filtered when the following conditions are satisfied.

1. EBF_e_ ≥ C_1_, where C_1_>1 is a cutoff set *a priori* by the user, with higher values being more stringent (more conservative filtering). C_1_=100 by default. This condition encodes sufficient evidence that (branch,site) belongs to the error class.
2. EBF_s_ ≥ C_2_, where C_2_>1 is a cutoff set *a priori* by the user, with higher values being more stringent (more conservative filtering). C_2_=20 by default. This condition encodes sufficient evidence that the error class is strongly preferred to the positive selection class.

When the branch being filtered is a leaf, only the corresponding (observed) codon is masked. When the branch being filtered is an internal branch, there is no corresponding observed codon, and all of the observed codons in the smaller of the two tree splits defined by the branch are masked. A third user controlled threshold 0<C_3_≤1 (default 0.4) is used to mask the entire site (as potentially unreliable) if more than C_3_ fraction of observed codons have been masked. Figure 5 illustrates the filtering procedure on a few key examples.

### Implementation and performance.

BUSTED-E is implemented in HyPhy versions 2.5.53 or later, as well as on the Datamonkey web application (Weaver et al. 2018); versions of BUSTED-E are different between HyPhy versions, and all of the analyses in the paper were done with 2.5.60, 2.5.61, or 2.5.62. Supplying --error-sink Yes to a hyphy busted call turns on the error sink component. Because BUSTED-E is a mixture random effects model, its parameter estimation using direct optimization (as done here) is sensitive to starting conditions. We devised a multistage model fitting procedure which attempts to identify good starting points to speed up BUSTED-E convergence and improve run-to-run reproducibility.

1. Fit the nucleotide GTR model to obtain initial estimates of branch lengths and nucleotide bias parameters $\left(\theta_{ij}\right)$.
2. Fix all estimates from phase 1, and estimate mean ω_0_ value (or values if there is more than one branch partition) using the MG94xREV codon model.
3. Use parameter estimates from phase 2 as the starting point, to re-estimate branch lengths, nucleotide bias parameters, and the mean ω_0_ value(s) under the full MG94xREV codon model.
4. Fix parameter estimates from phase 3. Generate K (=250 by default) Latin Hypercube random samples of ω distributions with means constrained to ω_0_ estimates from phase 3, and ɑ distributions with means constrained to 1.
5. Compute the phylogenetic likelihood function on the K initial points, select P (=5 by default) best scores.
6. Perform “quick-and-dirty” ω and ɑ distribution optimizations from using the P starting points from phase 5. The optimizations are done using the Nedler-Mead simplex algorithm (Nelder and Mead 1965), with the stopping criterion that consecutive iterations fail to improve the log likelihood score by at least $max\left(0.5,\;-10^{-4}\;log\;L(\right.phase\;3))$.
7. Select the estimates yielding the best likelihood score as the initial condition for the full direct optimization of the BUSTED model.

Additionally, if both BUSTED and BUSTED-E are fitted to the same alignment, we first run BUSTED, and add the parameter estimates obtained from BUSTED as 50% of the starting points candidate for step 4 in the fit for BUSTED-E, with the values of the error-sink drawn class drawn randomly.

### Simulated null data analysis.

We parametrically generated simulated alignments under the BUSTED (+S) model, using six empirical alignments as guides. Specifically, we fitted the BUSTED (+S) model with 2 or 3 rate classes to the biological alignments previously analyzed for evidence positive selection in multiple papers. The alignments included different numbers of sequences, varied in length, divergence, nucleotide composition, nucleotide substitution biases, and the inferred site-to-site distribution of synonymous rates (Table 13). For each empirical dataset, we generated 100 replicates under one of 10 selective profiles, summarized in Table 14, yielding 6,000 total simulated alignments. We then ran BUSTED and BUSTED-E on each replicate and tabulated the results, focusing on the power to detect selection, biases in rate estimates, and the rate at which BUSTED is rejected in favor of BUSTED-E.

### Empirical data analysis.

There are well over ten thousand papers which have used codon-based models to investigate natural selection with *d*_N_/*d*_S_ methods. Through a literature search, we identified four published *d*_N_/*d*_S_ based analyses of natural selection using coding sequences which also made the underlying sequence alignments publicly available, as supplementary information or via data repositories such as Zenodo, DataDryad or FigShare. Ours is not an exhaustive list of such studies, because the compilation of such a list, together with an attendant comprehensive re-analysis of millions of alignments is beyond the scope of our study. For each selected reference, we executed the following workflow.

1. If phylogenetic trees were not included with the data, infer trees alignment-by-alignment using raxml-ng (GTR+G+I model, all other settings at default values). Otherwise, provided trees (gene- or species-, depending on the study) were used.
2. Run BUSTED and BUSTED-E, including synonymous rate variation and setting N=3 rate classes (+1 for BUSTED-E) on each alignment.
3. Apply the BUSTED-E filter to the alignments, and run BUSTED and BUSTED-E on these filtered data.
4. Compare inference results (positive selection identified), inferred ω distributions, and perform descriptive analyses of filtering results.
5. Depending on the analyses performed in the original paper, we explore the effects of alignment filtering on comparable analyses.

All original and filtered multiple sequence alignments and trees are available from https://github.com/veg/pub-data

### Interaction with other filtering methods

To understand the type of alignment errors detected by BUSTED-E filtering, we explored how traditional alignment filtering methods interact with BUSTED-E. We selected two highly cited “conventional” alignment filtering methods: BMGE (Criscuolo and Gribaldo 2010) and HMMCleaner (Di Franco et al. 2019) to use in conjunction with BUSTED-E. BMGE utilizes a sliding window approach to detect regions of high entropy, while HMMCleaner generates an alternative sequence aligned with the original sequence to find regions of low similarity. Using data from Schulz and Sackton (2019), we filtered the alignments with each of the conventional filtering methods and used both BUSTED and BUSTED-E to explore the differences in EDS detection rate.

## Discussion.

In the early days of comparative evolutionary analyses, finding a positively selected gene (e.g., HIV-1 envelope or mammalian immune genes) was rare and remarkable, because data were sparse and statistical methods were crude and insensitive, requiring truly powerful signals (Hughes and Nei 1989; Lamers et al. 1993). Today, thousands of sequenced genomes are available, and statistical methods have been made much more sensitive. As a result, analyses often find that the majority of genes may have been subject to episodes of positive selection (Sackton 2020). The imprimatur of “positive selection” has lost its luster. Researchers must further refine prolific candidate lists of selected genes to confirm that the findings are robust and meaningful. Multiple methods are applied, and only genes where all methods agree are tagged as selected. Complex data filtering procedures are devised and run to reduce the influence of outliers and poor quality sequences. Follow-up analyses, such as functional enrichment are performed, to check the results using extrinsic attributes.

Alignment filtering and curation have received significant attention in the field with tools such as GBLOCKS (Castresana 2000), ALISCORE (Misof and Misof 2009), TrimAl (Capella-Gutiérrez et al. 2009), BMGE (Criscuolo and Gribaldo 2010), Zorro (Wu et al. 2012), GUIDANCE (Sela et al. 2015), HMMclean (Di Franco et al. 2019), HoT (Landan and Graur 2007). However, since the ground truth, i.e., what is the correct homology and alignment, is not known in practice, all approaches must rely on reasonable but ultimately unverifiable assumptions about what constitutes error. These assumptions are usually not integrated with or informed by downstream analyses that consume MSAs. This creates the potential for errors that bias downstream analyses to sift through the filters. As we demonstrated throughout the manuscript, using published data where the authors made good-faith reasonable efforts to filter alignment errors with various tools, numerous “obvious to the eye” (Figure 1) errors make it through the filtering process. Sensitive branch-site dN/dS models like BUSTED have little trouble pinpointing such errors with aberrant parameter estimates: very high dN/dS values affecting small fractions of the alignment. Leveraging this simple intuition to define *ad hoc* error-regions for dN/dS parameter estimates (dN/dS ≥ 100, weight ≤ 1%) turns out to be quite effective at reducing the rate of positive selection detection, with the implication that the majority of detected signal in many large scale screens is due to false positives.

For a typical practitioner, dN/dS based tests of selection are “black boxes”, and the source of signal, or the influence of outliers is difficult to assess (Spiess et al. 2024), especially if applied at scale. Intuitively, inference of selection is more robust if it is based on multiple substitutions across multiple sites. As large scale sequencing projects ramp up species coverage, including many (divergent) species in selection analysis increases the chances of mis-alignment and other error. Given enough data with some errors, and the challenges of catching such errors via automated procedures, every gene in the genome would be inferred under positive selection on at least one branch eventually. This, of course, is biologically meaningless, and makes selection analyses useless as a binary discriminative tool.

On the other hand, even for moderate sized datasets analyzed here, BUSTED-E error-correction framework improves downstream biological inference by reducing false positive signals due to confounders (protein length), and improving functional relevance, thus enhancing our ability to characterize genome-wide diversification pressures accurately.

We suggest that whenever researchers consider using dN/dS analyses, such as BUSTED especially in high-throughput settings, an error-absorbing framework like BUSTED-E be used routinely. In genome-wide scans, especially those involving a large number of species, an automated method to account for sequencing and alignment error will be crucial, lest all of the signal be swamped with residual error. BUSTED-E is not meant to be a primary filter for gross or large-scale errors, better handled by other tools, but rather as a post-filtering filter, as was done here. To guard against the loss of power, we recommend using model averaged approaches. While alignment masking is not necessary for BUSTED-E to mitigate errors during selection screens, its ability to perform such masking is useful prior to utilizing other tools which are not equipped with error filtering (Kosakovsky Pond and Frost 2005; Yang 2007; Murrell et al. 2012).

BUSTED-E is at its heart an *ad hoc* method. On the one hand it clearly finds many obvious errors missed by routine filtering, reduces EDS detection rate to credible levels, and results in more sensible functional annotation of selected genes. On the other hand, the error component that we introduce is heuristic and phenomenological, albeit tunable. Other settings for the error component class, or even multiple error classes will influence what is considered an error. Similarly, the choice of baseline substitution model has a major impact on error filtering as well. We view BUSTED-E as a practical and necessary first solution to dealing with pervasive alignment contamination to be refined and improved. Because we do not know the distribution and nature of sequencing and alignment errors, we expect that BUSTED-E will fail to detect many real errors, and also erroneously flag some biological variation as error. Power loss, possibly significant for some selection regimes, is also a concern, although our simulations show that given even moderate selection signal, this power loss is minimal. Power loss can be mitigated with model averaging.

Despite these limitations, BUSTED-E is a robust and scalable “drop-in” solution for improving the accuracy of evolutionary selection analyses in the presence of alignment errors, contributing to a more nuanced understanding of natural selection and adaptive evolution.

## Supplementary Material

Supplement 1

Supplement 2

Supplement 3

## Figures and Tables

**Figure 5.**
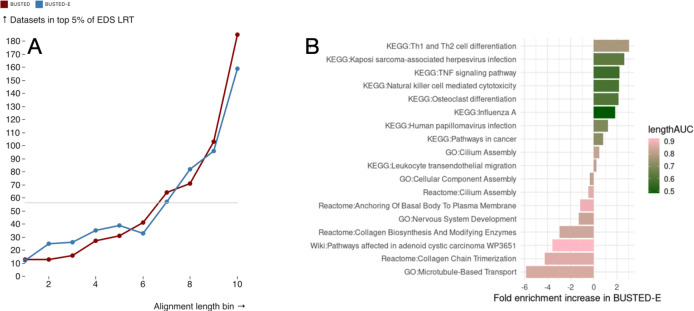
(A) Number of datasets that rank in the top 5% of the likelihood ratio test statistic (LRT) distribution for EDS, binned by the quantiles of alignment length distribution (shortest to longest); the grey horizontal line shows a uniform distribution reference (B) Enrichment/depletion analysis using top 5% LRT genes (BUSTED-E) as the foreground set, at FDR ≤ 0.2. Each bar is colored by the AUC of a simple predictive model which uses alignment length to predict BUSTED LRT model ranking (see text).

**Figure 5.**
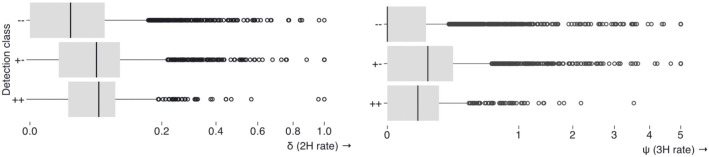
Point estimates of multiple-nucleotide substitution rates (under BUSTED-MH) stratified by BUSTED/BUSTED-E (without MH support) EDS classification at p≤0.05, e.g. +−: detected as EDS by BUSTED, but not by BUSTED-E.

**Figure 5.**
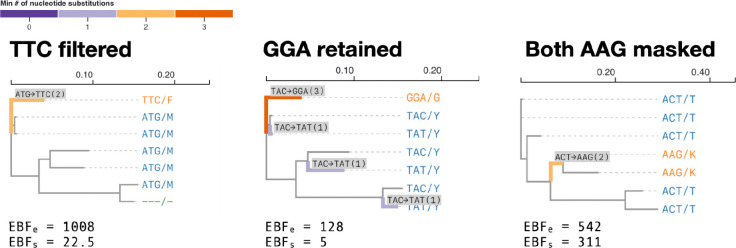
Examples of codon sites filtered and retained by the heuristic filtering procedures based on the empirical Bayes factors for error class membership and for positive selection.

**Table 1.** Two genes from Schultz and Sackton (2019) found to be subject to episodic diversifying selection (EDS) by BUSTED-S, along with the maximum likelihood estimates of the ω ratio distribution, and corresponding estimates from BUSTED-E.

Gene	p-value	ω_1_ (weight)	ω_2_ (weight)	ω_3_ (weight)	ω_E_ (weight)
**BUSTED-S**					
37524/HAUS1	0.00014	0.44(15.7%)	0.54 (55.0%)	2.27 (29.3%)	-
24389/PXDNL	0.00002	0.0 (0.3%)	0.068 (99.7%)	6070 (0.01%)	-
**BUSTED-E**					
37524/HAUS1	0.006	0.29 (22.4%)	0.54 (37.0%)	1.84 (40.5%)	100 (0.14%)
24389/PXDNL	0.50	0.0 (9.4%)	0.065 (89.4%)	1.0 (1.2%)	10000 (0.01%)

**Table 2.** Features of the four previously published genomics datasets.

Study PMID	Alignments			Tree length, subs/site	Gaps per		Alignment	MSA filtering
	Count	Seqs.	Codons		Column	Sequence/ 1k codons				
20333182	9404	7 [7:7]	469 [120:1850]	0.41 [0.17:0.86]	0.18 [0.0:1.0]	25.3 [0.0:141.9]	P2C / Darwin	Manual
25716091	4981	6 [5:6]	511 [154–1898]	0.21 [0.09:0.50]	0.34 [0.0:1.17]	58.1 [0.0:203.0]	OrthoMam v. 6	Gblocks
29953708	4248	15 [15:15]	246 [81:832]	0.15 [0.08–0.44]	0.00 [0.00:0.00]	0.0 [0.00:0.00]	Codons/PRANK	Guidance, manual (sliding window). Manual checking for MNM
30620335	11267	39 [24:44]	387 [88:1807]	1.3 [0.5–3.9]	0.73 [0.00:3.41]	19.4 [0.0:97.0]	P&C/MAFFT, PRANK	Custom (gap fractions & patterns; sliding window)

For all per-alignment quantities (sequences, codons, tree length, gaps per) we report the median value and the [2.5% : 97.5%] range. **Gaps per** statistics are based on the presence of fully gapped (---) codons; fully or partially ambiguous codons (e.g. ACN) are excluded. Gaps per sequence are further normalized per 1000 codons of sequence length. **Tree length** is the cumulative branch length (expected substitutions/nucleotide) under the MG94xREV model. Abbreviations for the **Alignment** column are as follows. *P2C:* translated protein sequences are aligned, then mapped back to codon sequences; *P&C:* protein based homology and filtering, codon-level alignment; *MNM* : multi-nucleotide mutations.

**Table 3.** Empirical data EDS analyses.

Data	EDS detection rate (reject the null at p≤0.05)			Original study selection detection rate	Rate of support for BUSTED-E (p≤0.05)	Means/medians of rate parameter estimates				Mean % error under the -E model (fraction of 0s)
						ω>1		% ω>1		
	Std.	-E	MA			Std.	-E	Std.	-E	
**PMID 20333182 (**Schneider et al. 2009)										
Original	0.39	0.12	0.21	0.009–0.233^1^	0.062	∞/3.6	∞/2.0*	4.8/1.8	5.7/2.3*	0.09 (63.9%)
Filtered	0.27	0.13	0.22		0.0032	∞/2.1	∞/1.9	5.8/2.4	5.8/2.5	0.01 (94.7%)
**PMID 25716091 (**Nguyen et al. 2015)																					
Original	0.62	0.32	0.43	N/A^2^	0.2	∞/21	∞/8.5*	4.2/1.9	4.1/1.8	0.21 (39.3%)
Filtered	0.32	0.15	0.22		0.02	∞/4.6	∞/3.4*	3.1/0.94	3.1/0.88	0.044 (92.6%)
**PMID 29953708 (**Wu et al. 2018)																					
Original	0.091	0.012	0.034	~0.01–0.02^3^	0.0054	∞/4.1	∞/3.2*	3.6/0.49	3.8/0.48	0.02 (88.4%)
Filtered	0.067	0.013	0.032		0.0047	∞/3.6	∞/3.1*	3.6/0.46	3.7/0.46	0.0061 (96.6%)
**PMID 30620335 (**Shultz and Sackton 2019)																					
Original	0.42	0.079	0.17	0.21 –0.73^4^	0.069	∞/7.8	∞/3.7*	1.3/0.13	1.5/0.15	0.012 (63.2%)
Filtered	0.26	0.078	0.16		0.013	∞/7.9	∞/3.4*	1.3/0.12	1.3/0.12	0.003 (91.6%)

BUSTED-S (Std.) and BUSTED-E (-E) performance on empirical data sets; both original and filtered using BUSTED-E (see text). MA: model average.

*: estimates between BUSTED-E and BUSTED have a Mann-Whitney test p < 0.0001.

(1) Per lineage branch-site tests using PAML at FDR≤0.05 following the Rom correction.

(2) No direct dN/dS ≠ 1 tests were done.

(3) FDR≤0.01 corrected clade branch-site tests using PAML for different clades.

(4). p ≤ 0.05 for different PAML model pairs and the original BUSTED method. The study used a 0.14 rate by requiring unanimous method consent.

**Table 4.** Log likelihoods and estimated ω rate distributions under null and alternative EDS testing hypotheses with BUSTED and BUSTED-E for the SUGP2 (10004) gene.

Model	Log (L)	ω_1_ (weight)	ω_2_ (weight)	ω_3_ (weight)	ω_E_ (weight)
BUSTED Alternative	−27171.8	0.00 (1.7%)	0.15 (98.2%)	28.23 (0.12%)	N/A
BUSTED Null	−27185.9	0.11 (13.7%)	0.12 (81.7%)	1.00 (4.6%)	N/A
BUSTED-E Alternative	−27171.8	0.00 (1.7%)	0.15 (98.2%)	28.23 (0.12%)	* (0.0%)
BUSTED-E Null	−27173.2	0.05 (8.5%)	0.15 (89.7%)	1.00 (1.8%)	100 (0.05%)

(*) rate not identifiable, because the corresponding weight is estimated to be 0.

**Table:** 

Model	Log (L)	ω_1_ (weight)	ω_2_ (weight)	ω_3_ (weight)	ω_E_ (weight)
BUSTED Alternative	−14556.5	0.009 (95.9%)	0.013 (4.1%)	584.3 (0.02%)	N/A

**Table 6.** Classification of alignments with discordant EDS results at p < 0.05. Discordant results were categorized into five types, described in the text, based on BUSTED and BUSTED-E results.

Dataset	Discordant N (%)	Type 1 (%)	Type 2 (%)	Type 3 (%)	Type 4 (%)	Type 5 (%)
***BUSTED vs BUSTED-E***						
20333182	2577 (27.4)	309 (12.0)	793 (30.8)	243 (9.4)	429 (16.6)	803 (31.2)
25716091	954 (19.3)	365 (38.3)	149 (15.6)	75 (7.9)	109 (11.4)	256 (26.8)
29953708	339 (8.0)	3 (0.9)	145 (42.8)	78 (23.0)	59 (17.4)	54 (15.9)
30620335	3814(33.9)	262 (6.9)	1181 (31.0)	545 (14.3)	923 (24.2)	903 (23.7)
***BUSTED vs Model Averaged (MA)***						
20333182	1727 (18.4)	308 (17.8)	207 (12.0)	166 (9.6)	423 (24.5)	623 (36.1)
25716091	725 (14.7)	359 (49.5)	33 (4.6)	42 (5.8)	107 (14.8)	184 (25.4)
29953708	243 (5.7)	2 (0.8)	68 (28.0)	69 (28.4)	59 (24.3)	45 (18.5)
30620335	2763 (24.5)	256 (9.3)	486 (17.6)	453 (16.4)	888 (32.1)	680 (24.6)

**Table 7.** The two example genes from Table 1 passed through the BUSTED-E filtering procedure and reanalyzed for evidence of EDS.

Gene	p-value	ω_1_ (weight)	ω_2_ (weight)	ω_3_ (weight)	ω_E_ (weight)
**BUSTED-S**					
37524/HAUS1	0.034	0.00 (18.9%)	1.00 (44.2%)	1.45 (36.9%)	-
24389/PXDNL	0.26	0.006 (52.2%)	0.13(47.8%)	4.65 (0.06%)	-
**BUSTED-E**					
37524/HAUS1	0.034	0.013 (19.6%)	1.00 (40.8%)	1.43 (39.7%)	* (0.00%)
24389/PXDNL	0.50	0.00 (53.6%)	0.15(46.4%)	3.58 (0.00%)	>6000 (0.001%)

**Table 8.** BUSTED-E filtering procedure statistics.

Study PMID	Unaffected alignments (%)	Masked codons per			As fraction of total gaps, %
		Alignment	Site	1K of sequence	
20333182	6263 (66.6)	11 [1.0–79.1]	0.0027 [0.00022–0.011]	2.7 [0.22–11]	7.7 [0.42–96]
25716091	3172 (63.7)	9 [1.0–71.0]	0.0028 [0.00026–0.014]	2.8 [0.26–14]	5.3 [0.31–100]
29953708	3771 (88.8)	9 [1.0–52.7]	0.0023 [0.00013–0.011]	2.3 [0.13–11]	100 [100–100]*
30620335	7245 (64.3)	32 [1.0–262.0]	0.0019 [7.5e-05–0.0095]	1.9 [0.075–9.5]	9.3 [0.24–100]

Unaffected alignments are those where no filtering is deemed necessary. All the following statistics are computed only over those alignments where some filtering was done. **Masked codons per** is the median [2.5% – 97.5%] number of codons masked per: alignment (raw counts), a single column (normalized), 1000 codons of sequence (normalized). **As fraction of total gaps**, refers to the median [2.5% – 97.5%] percentage of the gaps in the filtered alignment that are due to those added by filtering (the others were already there).

* Input datasets for this reference contained no gaps.

**Table 9.** ***Top:*** Upset plot showing the overlap of genes under EDS detected by BUSTED on the original data from PMID 30620335 (Unfiltered), or data filtered with BMGE or HMMCleaner, and by BUSTED-E on the original data. ***Bottom:*** The effects of various data filtering approaches on BUSTED and BUSTED-E performance; the “original data” row also includes filtering metrics using BUSTED-E.

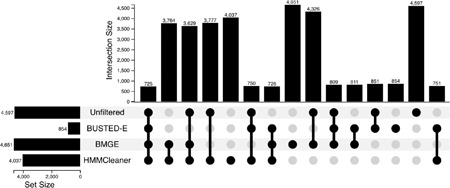					
Filtering method	EDS detection rate (reject the null at p<0.05)		Average runtime (seconds)	% of genes with at least one site filtered	Average fraction of sites filtered
	BUSTED	BUSTED-E			
Original data	0.4175	0.079	454 (BUSTED) 658 (BUSTED-E)	60.8% (BUSTED-E)	1.964 e-3 (BUSTED-E)
HMMCleaner	0.367	0.047	716	4.28%	1.365e-3
BMGE	0.420	0.059	540	61.3%	1.743e-4

**Table 10.** The two example genes from Table 1 re-analyzed with BUSTED/BUSTED-E with support for multiple nucleotide substitutions (Mh).

Model	p-value	ω_1_ (weight)	ω_2_ (weight)	ω_3_ (weight)	ω_E_ (weight)	δ (2-hit rate)	Ψ (3-hit rate)
**TLK2 gene (Type 1 discordant)**							
BUSTED	0.00	0.00 (0.4%)	0.18 (100%)	1534 (0.02%)	-	-	-
BUSTED-E	0.10	0.00 (0.7%)	0.17 (100%)	10.79 (0.1%)	2089 (0.01 %)	-	-
-MH	0.50	0.16 (40%)	0.18 (60%)	1.00 (0%)	-	0.03	0.14
-MH-E	0.50	0.16 (40%)	0.18 (60%)	1.06 (0%)	1480 (0.004%)	0.03	0.14
**SUGP2 (Type 2 discordant)**							
BUSTED	0.00	0.00 (1.7%)	0.15 (98.2%)	28.23 (0.1%)	-	-	-
BUSTED-E	0.12	0.00 (1.7%)	0.15 (93.2)	28.57 (0.1%)	* (0%)	-	-
-MH	0.50	0.15 (100%)	* (0%)	* (0%)	-	0.08	0.14
-MH-E	0.50	0.15 (100%)	* (0%)	* (0%)	* (0%)	0.08	0.14
**VEPH1 (Type 3 discordant)**							
BUSTED	0.00	0.11 (92.2%)	1.00 (7.8%)	207 (0.03%)	-	-	-
BUSTED-E	0.50	0.11 (92.1%)	1.00 (7.9%)	233 (0.03%)	* (0%)	-	-
-MH	0.50	0.12 (54.3%)	0.17 (42.4%)	1.00 (3.3%)	-	0.04	0.18
-MH-E	0.50	0.12 (54.4%)	0.17 (42.3%)	1.00 (3.3%)	* (0%)	0.04	0.18
**BSDC1 (Type 4 discordant)**							
BUSTED	0.00	0.00 (50.3%)	0.36 (49.7%)	>1000 (0.02%)	-	-	-
BUSTED-E	0.50	0.00 (50.0%)	0.36 (50.0%)	* (0%)	>1000 (0.02%)	-	-
-MH	0.50	0.12 (85.3%)	0.49 (14.7%)	* (0%)	-	0.04	0.05
-MH-E	0.50	0.08 (78.3%)	0.54 (21.6%)	* (0%)	>1000 (0.02%	0.00	0.00
**GSTK1 (Type 5 discordant)**							
BUSTED	0.01	0.33 (95.2%)	1.00 (4.2%)	13.24 (0.63%)	-	-	-
BUSTED-E	0.29	0.33 (95.1%)	0.53 (3.3%)	4.81 (1.6%)	165 (0.05%)	-	-
-MH	0.50	0.16 (39.3%)	0.52 (60.7%)	* (0%)	* (0%)	0.04	0.15
-MH-E	0.50	0.16 (43.5%)	0.55 (56.1%)	2.9 (0.35%)	134 (0.04%)	0.02	0.11

**Table 11.** Key model parameters controlling rate variation.

Parameter	Role	Variation	Distribution
**a(s)**	Relative synonymous rate at a site	Independent among sites.	M-bin unit mean general discrete distribution
ω**(s,b)**	Non-synonymous to synonymous substitution rates ratio on a branch at a site	Independent among sites and branches.	N-bin general discrete distribution. $\omega_{1}\leq \omega_{2}\ldots \leq 1\leq \omega_{N-1}$ $1\ll E\leq \omega_{N}$

**Table 12.** Parameterization of entries in the instantaneous substitution rate matrix.

Class	Substitution rate	Example
**One-nucleotide synonymous**	$\alpha \theta_{ij}\pi_{j}^{k}$	AAA to AAG: $\alpha \theta_{AG}\pi_{G}^{3}$
**One-nucleotide non-synonymous**	$\alpha \omega \theta_{ij}\pi_{j}^{k}$	CAA to CTA: $\alpha \omega \theta_{AT}\pi_{T}^{2}$
**Multi-nucleotide substitution**	**0**	CCC to TTT

**Table 13.** The six empirical alignments used as stencils for simulations under the BUSTED model.

Dataset	N	S	T	Composition, % (A, C, G, T)	Relative nucleotide substitution rates (AC, AG=1, AT, CG, CT, GT)	Mean ω	CoV synonymou s rates	Rate classes: a, ω
β-globin	17	144	2.24	20.8, 26.1,29.2, 23.9	0.71,0.36, 0.57, 1.59, 0.51	0.24	0.35	2,2
Sperm lysin	25	134	2.54	28.5, 21.3, 24.9, 25.3	0.85, 0.45, 0.66, 0.70, 0.26	0.94	0.85	3,2
Drosophila adh	23	254	1.36	23.3, 28.6, 25.4, 22.7	0.77, 0.51,0.79, 2.29, 0.48	0.09	0.34	3,2
Hepatitis D	33	196	1.76	30.1,22.3, 36.9, 10.8	0.44, 0.56, 0.29, 1.52, 0.21	0.42	0.84	3,3
Echolocating bats *SWS1*	33	286	1.10	16.4, 30.5, 26.0, 27.1	0.19, 0.07, 0.13, 0.73, 0.14	0.22	0.30	2,3
*vwl*	62	392	5.02	20.7, 30.3, 30.9, 18.1	0.32, 0.21,0.38, 1.25, 0.19	0.19	0.42	3,3

N = number of sequences; S = number of codons; T = total tree length, subs/nucleotide site; CoV = coefficient of variation.

**Table 14.** Parametric BUSTED simulation scenarios (no error).

Scenario	ω_+_	Pr (ω = ω_+_)
1. Neutral Evolution	1.0	0.10
2. Very weak EDS	1.5	0.05
3. Weak EDS	2.5	0.05
4. EDS, very small fraction	5.0	0.01
5. EDS, small fraction	5.0	0.025
6. EDS	5.0	0.05
7. EDS, large fraction	5.0	0.10
8. Strong EDS	10.0	0.05
9. Very high ω	50.0	0.025
10. Error only	100.0	0.01
