# Supplementary material for: Minus the Error: Estimating dN/dS and Testing for Natural Selection in the Presence of Residual Alignment Errors
Source: bioRxiv. 2024 Nov 15:2024.11.13.620707. Preprint. [Version 1] doi: 10.1101/2024.11.13.620707 (PMC11601313; doi:10.1101/2024.11.13.620707)
Supplement: Supplementary file 1 [file media-1.pdf]

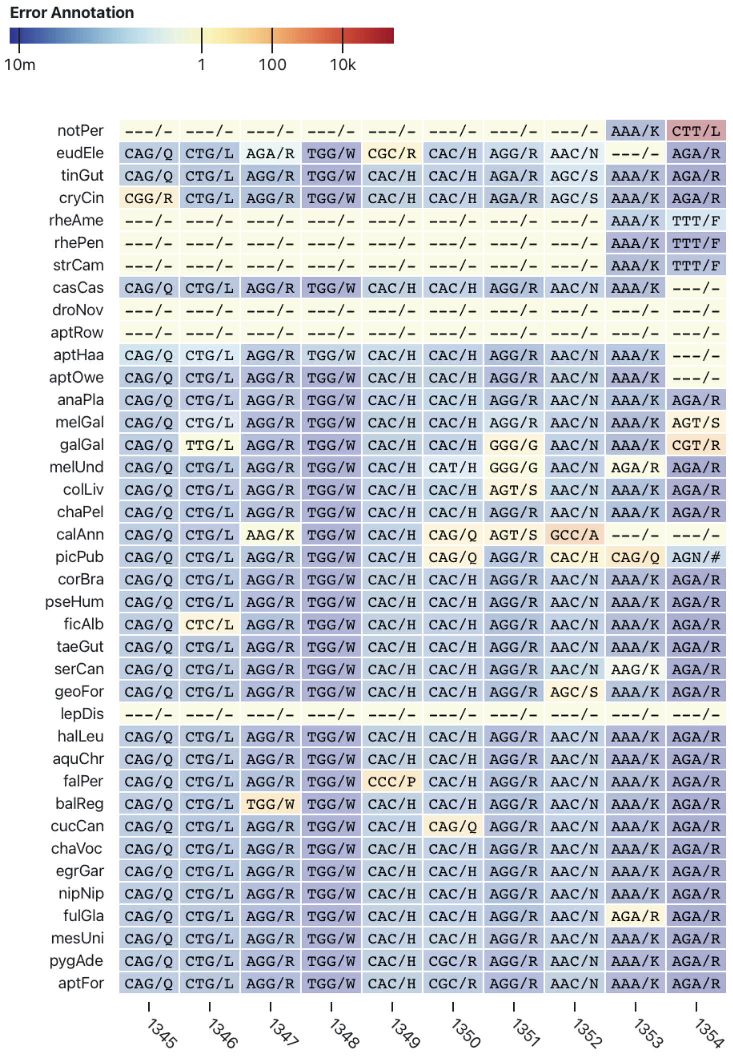

Figure S1 Apparent local misalignment at the 3’ end of the PXDNL gene from Shultz and Sackton. The color palette indicates empirical Bayes factor support for assignment to the error class.
