# Supplementary material for: Minus the Error: Estimating dN/dS and Testing for Natural Selection in the Presence of Residual Alignment Errors
Source: bioRxiv. 2024 Nov 15:2024.11.13.620707. Preprint. [Version 1] doi: 10.1101/2024.11.13.620707 (PMC11601313; doi:10.1101/2024.11.13.620707)
Supplement: Supplementary file 2 [file media-2.pdf]

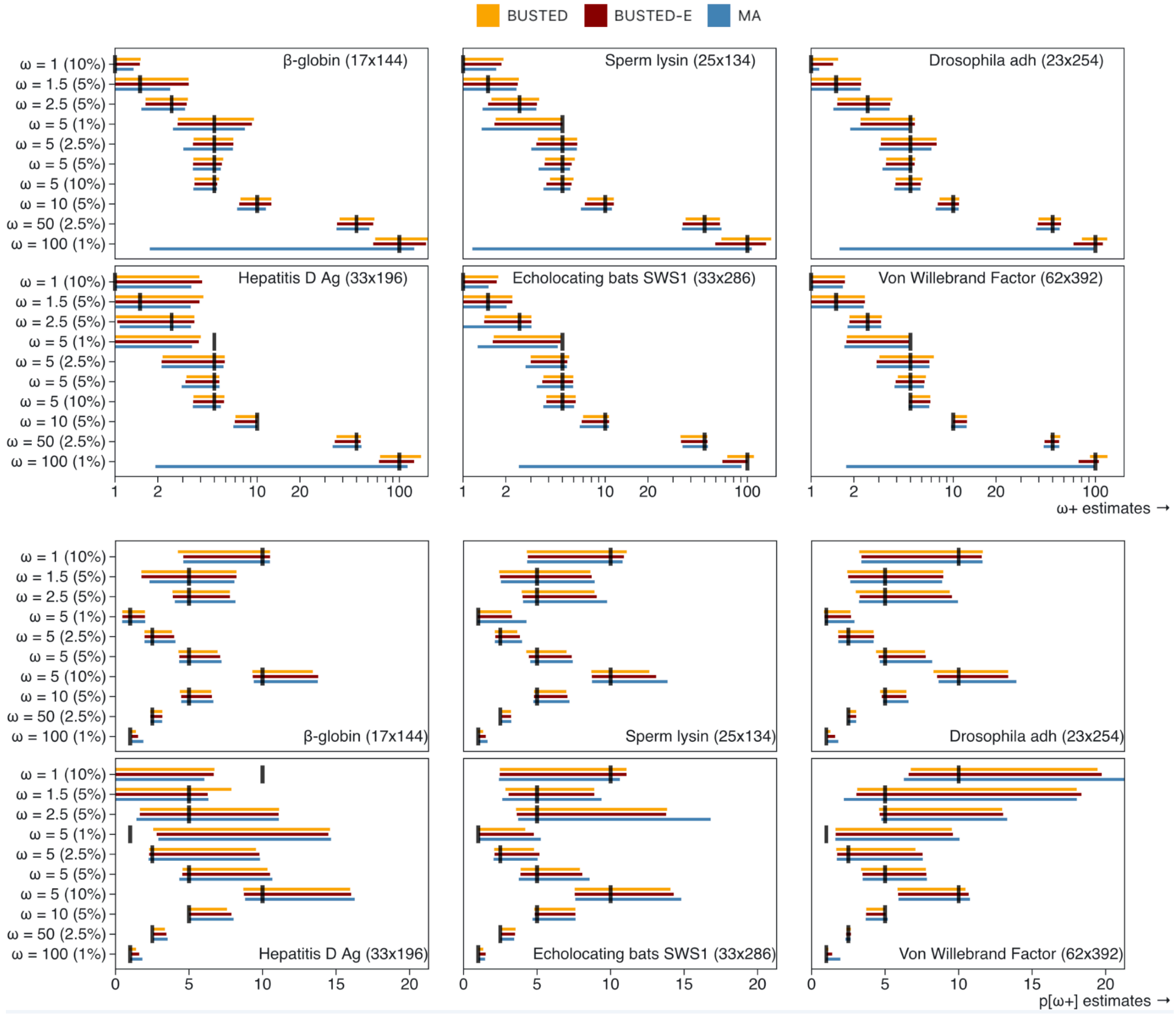

Figure S2. Interquartile ranges for key parameter estimates obtained from simulated data. Horizontal ticks show the values used for simulations. The top two rows show dataset specific estimates of the positive selection ω+ component, and the bottom two rows, the fraction assigned to ω+, measured as a percentage.
