# Supplementary material for: Minus the Error: Estimating dN/dS and Testing for Natural Selection in the Presence of Residual Alignment Errors
Source: bioRxiv. 2024 Nov 15:2024.11.13.620707. Preprint. [Version 1] doi: 10.1101/2024.11.13.620707 (PMC11601313; doi:10.1101/2024.11.13.620707)
Supplement: Supplementary file 3 [file media-3.pdf]

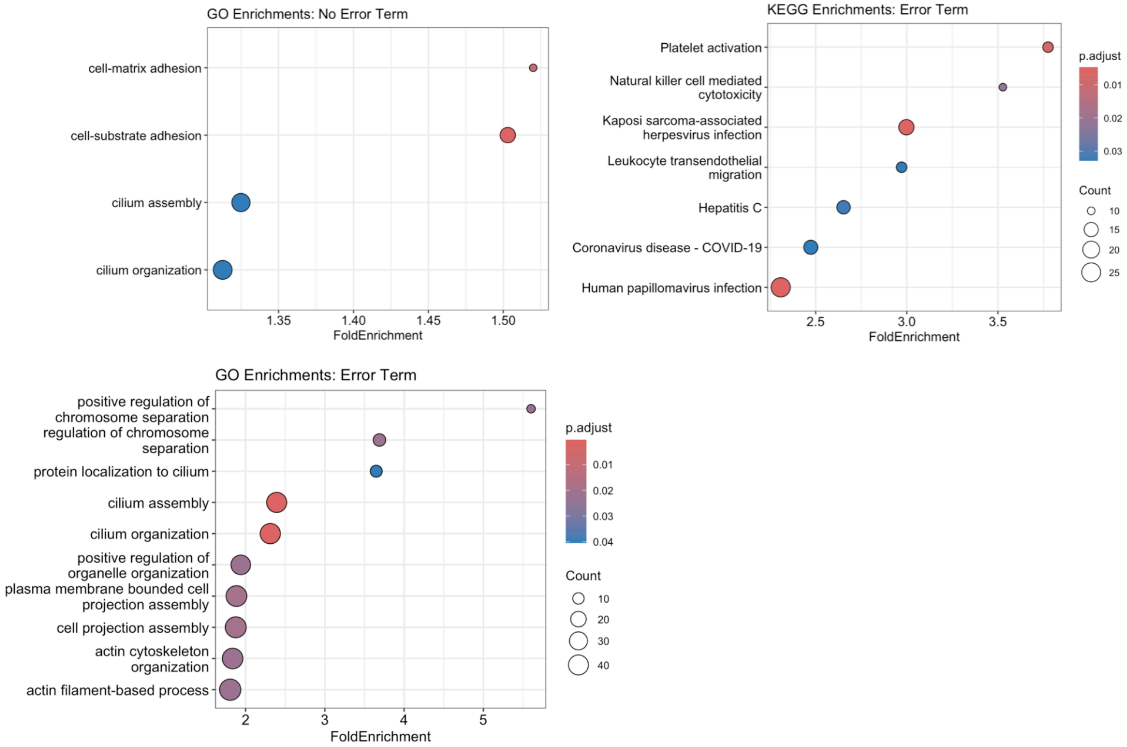

Figure S3 GO and KEGG pathways enrichment analysis (FDR ≤ 0.2) for genes found to be under selection at p ≤ 0.05 with BUSTED and BUSTED-E. Note that there were no significantly enriched KEGG pathways when using BUSTED (no error term).
